# Ocular Filariasis in Human Caused by *Breinlia* (*Johnstonema*) *annulipapillata* Nematode, Australia

**DOI:** 10.3201/eid2701.203585

**Published:** 2021-01

**Authors:** Anson V. Koehler, Jennifer M.B. Robson, David M. Spratt, Joshua Hann, Ian Beveridge, Michael Walsh, Rodney McDougall, Mark Bromley, Anna Hume, Harsha Sheorey, Robin B. Gasser

**Affiliations:** University of Melbourne, Parkville, Victoria, Australia (A.V. Koehler, I. Beveridge, R.B. Gasser);; Sullivan Nicolaides Pathology, Brisbane, Queensland, Australia (J.M.B. Robson, M. Walsh, R. McDougall, M. Bromley, A. Hume);; Australian National Wildlife Collection, Commonwealth Scientific and Industrial Research Organisation, Canberra, Australian Capital Territory, Australia (D.M. Spratt);; Eastside Eye Specialist Care, Carindale, Queensland, Australia (J. Hann);; St. Vincent’s Hospital, Melbourne, Victoria, Australia (H. Sheorey)

**Keywords:** Australia, human, marsupial, ocular filariasis, zoonoses, Breinlia (Johnstonema) annulipapillata, nematodes, parasites

## Abstract

We report a human case of ocular filariasis, caused by a species of *Breinlia* nematode, from Queensland, Australia. Morphological and molecular evidence indicated that the nematode *Breinlia* (*Johnstonema*) *annulipapillata*, or a closely related taxon, likely transmitted from a macropodid marsupial host was involved, which might represent an accidental finding or an emerging zoonosis.

Filariasis of the eye is commonly caused by adults or larvae of the filarioid nematodes *Onchocerca volvulus, Loa loa*, and *Dirofilaria immitis* (*1*), although sporadic cases involving *Acanthocheilonema*, *Loaina* (*1*,*2*), or *Pelecitus* (*3*) nematodes have been reported. Filarioids in eyes are challenging to identify morphologically to genus or species, because often only single, immature worms of 1 sex are present, the worms are degraded, or both (*2*). Molecular tools can generally improve the identification of worms of the eyes (e.g., *Dirofilaria hongkongensis* [*4*]), even if only to genus (e.g., *Pelecitus* sp. [*3*]). In Australia, *D. immitis* nematodes have typically been the causative agent of ocular filariasis infection in humans; the prevalence of dirofilariasis in dogs was historically quite high (up to 64%) in the subtropical and tropical climes, such as around Brisbane (*5*). We report a human case of an ocular infection by a *Breinlia* sp. nematode commonly found in Australian marsupials and rodents. 

## The Study

**Figure 1:**
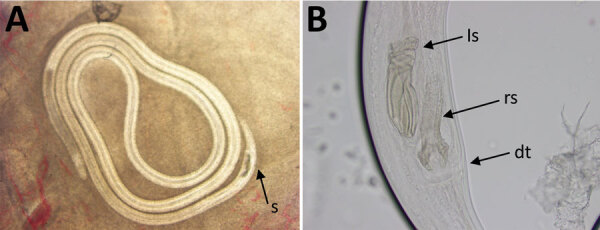
Identification of *Breinlia* sp. nematodes from a patient with ocular filariasis, Brisbane, Queensland, Australia, 2019. A) Photograph (in situ) of male *B.* (*Johnstonema*) *annulipapillata* nematode from the subconjunctiva, illustrating thick heavily sclerotized spicules (s). B) Right lateral view of male tail of *B.* (*J.*) *annulipapillata* nematode, illustrating left (ls) and right (rs) spicules; right spicules showed a bifurcated distal extremity (dt), a diagnostic character of the species.

In May 2019, a 73-year-old man in Brisbane, Queensland, Australia came to his optometrist with an irritated right eye and eyelid. Entropion was suspected, although the patient was unable to tolerate a thorough examination because of extreme irritation of the involved eye. He was referred to an ophthalmologist 3 weeks later; the eye was still irritated, but not grossly inflamed or red. Slit lamp examination revealed a motile nematode in the subconjunctiva (Figure 1; Video), which was extracted and fixed in neutral-buffered formalin. Initial morphological examination of the specimen revealed a male filarioid (17–20 mm long) with short, heavily sclerotized spicules; the right spicule had a bifid distal extremity, highly suggestive of *Breinlia* (*Johnstonema*) *annulipapillata* (Figure 1).

**Video:** Slit lamp video of live, coiled male filarial nematode, *Breinlia* (*Johnstonema*) *annulipapillata,* from the subconjunctiva of a human patient with ocular filariasis, Brisbane, Queensland, Australia, 2019.

The patient was born in Poland and immigrated to Melbourne in 1969, where he spent his working life before retiring to Brisbane in 2005. He had no pets or close contact with animals. His only recent travel was to the Gold Coast and to an island in Moreton Bay, both near Brisbane. The patient had no noteworthy medical history apart from hyperthyroidism, which was well controlled. C-reactive protein (CRP) and full blood count (FBC) test results were within reference ranges, with no eosinophilia, and results of filarial serologic testing (IgG enzyme immunoassay using antigen Bm14) were negative. After the nematode was removed from the patient’s eye, symptoms resolved. No anthelmintic medication was prescribed.

We extracted genomic DNA from the formalin-fixed paraffin-embedded worm using a GeneRead DNA FFPE kit (QIAGEN, https://www.qiagen.com) and then subjected it to PCR, targeting the small subunit of nuclear ribosomal RNA gene (SSU), and a nested PCR, targeting the mitochondrial cytochrome c oxidase subunit 1 gene (*cox*-1) (Table; *6*). Known positive (*Onchocerca volvulus* DNA) and no-template controls were included. Amplicons were sequenced using an established protocol (*8*).

**Table:** Primer sequences used in PCR of the amplification regions of the SSU or *cox*-1 genes of *Breinlia* sp. nematodes from a patient with ocular filariasis, Brisbane, Queensland, Australia, 2019*

Designation	Primer pair	Oligonucleotide sequence, 5¢ → 3¢	Annealing temperature, °C (time)†	Expected size, bp	Reference
SSU					
1° PCR	F18ScF1	ACCGCCCTAGTTCTGACCGTAAA	58 (45 s)	830	(*6*)
	F18ScR1	GGTTCAAGCCACTGCGATTAAAGC			
*cox*-1					
1° PCR	FCo1extdF1	TATAATTCTGTTYTDACTA	52 (45 s)	970	(*6*)
	FCo1extdR1	ATGAAAATGAGCYACWACATAA			
2° PCR	COIintF	TGATTGGTGGTTTTGGTAA	52 (45 s)	650	(*7*)
	COIintR	ATAAGTACGAGTATCAATATC			

* *cox*-1, cytochrome *c* oxidase subunit 1; SSU small subunit of nuclear ribosomal RNA. †All PCRs used 35 cycles with an initial denaturation at 94°C for 5 min, all subsequent denaturation cycles were 30 s, and all extensions were 1 min.

We assessed the sequences (GenBank accession nos. MT752937 [*SSU,* 724 bp] and MT754705 [*cox*-1, 660 bp]) for quality and compared them with those available publicly. Because sequence data for *SSU*, *cox*-1, or both were publicly available for only 3 taxa of 24 known species of *Breinlia*—*B. mundayi* from the swamp wallaby (*Wallabia bicolor*); *Breinlia* sp. from a Leadbeater’s possum (*Gymnobelideus leadbeateri*); and *B. jittapalapongi* from an Asian house rat (*Rattus tanezumi*) —molecular identification was limited to these taxa. The *SSU* sequence (724 bp) obtained for the worm under investigation was 99% similar to those of *B. mundayi* (GenBank accession no. JF934735; 708/710 bp), *Breinlia* sp. from an opossum (GenBank accession no. MT731343; 711/712 bp), and *B. jittapalapongi* (GenBank accession no. KP760119; 656/665 bp). The *cox*-1 sequence (660 bp) obtained was 92% similar to that of *B. jittapalapongi* (GenBank accession no. KP760170; 553/604 bp) and 91% similar to that of *Breinlia* sp. from the opossum (GenBank accession no. MT724666; 601/659 bp); no *cox*-1 sequence was publicly available for *B. mundayi*.

**Figure 2:**
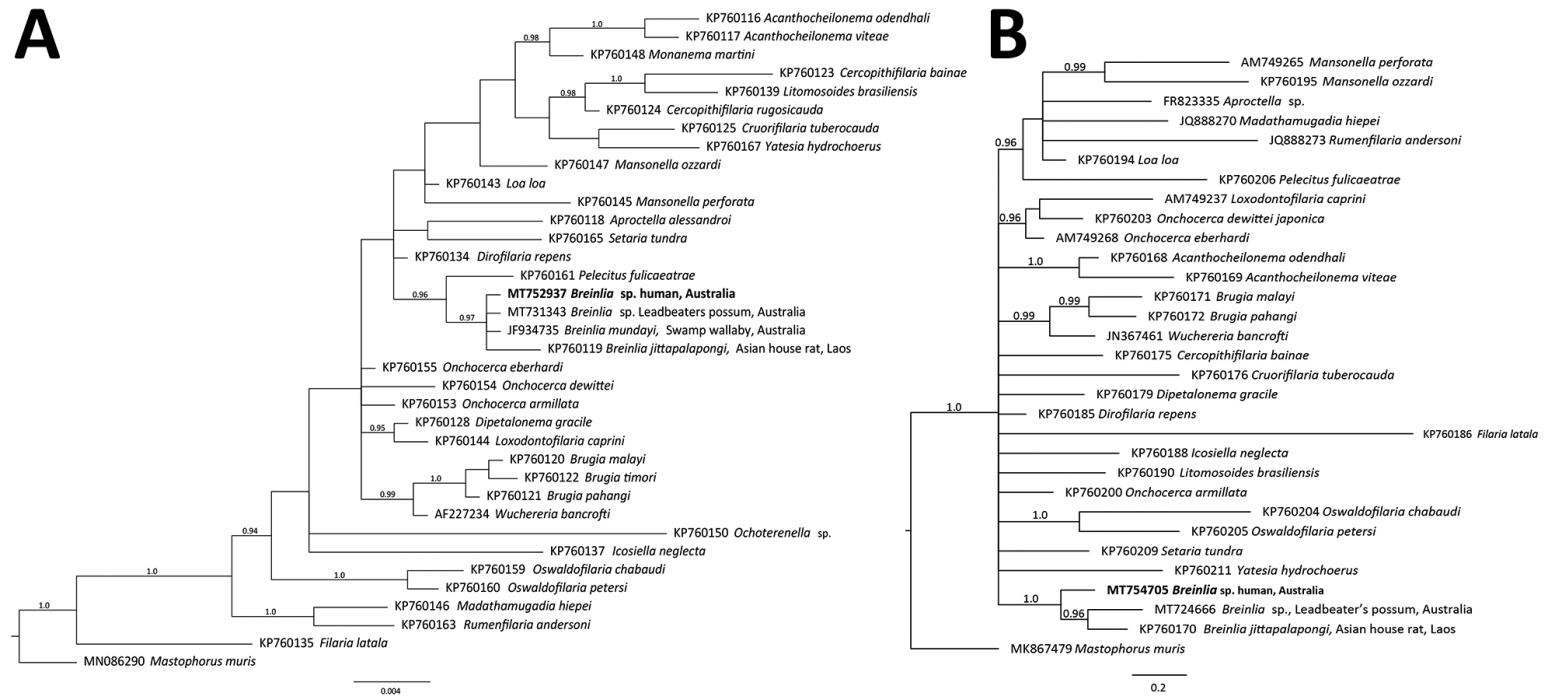
Relationship of the novel *Breinlia* sp. taxon (bold type), the nematode species recovered from the eye of a human patient with ocular filariasis, Brisbane, Queensland, Australia, 2019, with representative sequences from members of the family Onchocercidae based on phylogenetic analysis. A) Small subunit of nuclear ribosomal RNA gene; B) cytochrome oxidase 1 gene. Data were compiled using the Bayesian inference method. Branch support given in posterior probability. Respective sequences for *Mastophorus muris* (outgroup) were included in the analyses. GenBank accession numbers are provided. Scale bars represent expected substitutions per site.

The sequences obtained were aligned to those accessible publicly for 34 (*SSU*) or 29 (*cox*-1) species of filarioid and of *Mastophorus muris* (outgroup) (Figure 2). Aligned *SSU* and *cox*-1 sequence data were subjected to separate phylogenetic analyses using the Bayesian inference method (*8*), with nodal support values given as posterior probabilities. The resultant trees (Figure 2) revealed that the nematode under study is a member of the genus *Breinlia*, as it grouped with *Breinlia* from the opossum, *B. mundayi* (*SSU* only), and *B. jittapalapongi* with strong statistical support. Thus, this worm could be identified molecularly as a *Breinlia* sp.; it could not be identified to species because of the lack of sequence data for *Breinlia* spp. in public databases.

There are 5 reports of human intraocular filariasis from Australia: 4 suspected *D. immitis* cases from New South Wales, Queensland, and Victoria (*9*–*12*); and 1 *Dipetalonema* (*Acanthocheilonema*) *reconditum* case from Victoria (*13*). The short, heavily sclerotized spicules of this specimen, with a bifid distal extremity on the right spicule (Figure 1), indicated that it was neither of these taxa, but rather *B.* (*J.*) *annulipapillata*. This species occurs in a range of macropodid species, predominantly in northern Australia, although it is also found in swamp wallabies in the south. The nematodes of only other known species of the subgenus *Johnstonema*, *B.* (*J.*) *woerlei,* has much larger, heavily sclerotized spicules, but without a bifid extremity on the right spicule, and occurs in the short-eared rock wallaby (*Petrogale brachyotis*) in the Northern Territory (*14*).

Although no life cycles of subgenus *Johnstonema* nematodes are known, those of 4 species of the subgenus *Breinlia* are known and involve *Aedes* mosquitoes as intermediate hosts (*14*). The patient was probably been bitten by the intermediate host of this filarioid, possibly a mosquito, that had previously taken a blood meal from a macropodid and was carrying infective larval stages (L3s). Once in the patient, the L3s would have undergone 2 additional molts and established themselves in the eye and perhaps in other tissues throughout the body (although there was no evidence of infection elsewhere). Adult *Breinlia* nematodes are found predominantly in the peritoneal and pleural cavities of mammalian definitive hosts (*14*). However, other filarial nematodes have a tropism for the eye, and several cases have been reported of zoonotic filariasis of the eye relating to *Dirofilaria* sp. nematodes (*1*). *Breinlia* nematodes had not been found previously in humans, but *B. sergenti* nematodes has been recorded in the slow loris (*Nycticebus coucang*) in Southeast Asia (*14*). It is possible that ocular *Breinlia* infections may go undetected in humans, particularly in less conspicuous places than the eye, and may be more common than expected in areas where *Breinlia*-infected marsupials are prevalent.

## Conclusions

This human case of ocular filariasis caused by *Breinlia* sp. nematodes is highly unusual and was likely transmitted from a kangaroo or wallaby via a blood-feeding intermediate host, possibly a mosquito, to the patient. Microscopic identification of filarioids can be challenging, depending on their stage of development and sex, but fortuitously that was not the case here. Nevertheless, the use of the current molecular approach can be advantageous for generic or specific identification, provided that sufficient sequence data are available in public databases. We recommend that both morphological and molecular tools be used to attempt to achieve a specific diagnosis in cases of human ocular filariasis.
