# Supplementary figures and images for: Ocular Filariasis in Human Caused by Breinlia (Johnstonema) annulipapillata Nematode, Australia
Source: Emerg Infect Dis. 2021 Jan;27(1):297–300. doi: 10.3201/eid2701.203585 (PMC7774559; doi:10.3201/eid2701.203585)

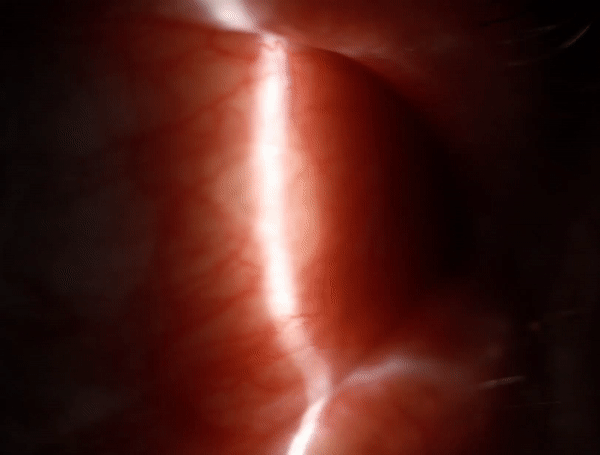

Supplement: Supplementary file 1 [file 20-3585-V.gif]
